# Ligand-receptor interactions of V-domain Ig-containing suppressor of T cell activation and programmed death-1 suppress the anticancer activities of T cells

**DOI:** 10.1016/j.iotech.2025.101533

**Published:** 2025-11-13

**Authors:** M. Abooali, X. Lei, I.M. Yasinska, S. Schlichtner, R. Hussain, G. Siligardi, T.-M. Gianga, S.M. Berger, D. Cholewa, B.F. Gibbs, E. Fasler-Kan, V.V. Sumbayev

**Affiliations:** 1 Medway School of Pharmacy, Universities of Kent and Greenwich, Chatham Maritime, UK; 2 The Ignaz Semmelweis Institute and Division of Infectious Diseases, Translational Medical Mycology Research Unit, Host-Fungal Pathogen Interaction Group, Medical University of Graz, Graz, Austria; 3 Beamline 23, Diamond Light Source, Didcot, UK; 4 Department of Paediatric Surgery, Children’s Hospital, Inselspital Bern, University of Bern, Bern, Switzerland; 5 School of Science, Psychology, Arts and Humanities, Computing, Engineering and Sport Science (SPACES), Canterbury Christ Church University, Canterbury, UK

**Keywords:** VISTA, PD-1, immunosuppression, T cells, cancer

## Abstract

**Background:**

V-domain immunoglobulin-containing suppressor of T cell activation (VISTA) is a unique multifunctional immune checkpoint protein, which can display both receptor and ligand properties. It plays a crucial role in the cancer immune evasion machinery operated by a wide range of human malignancies and may thus be considered as a potential target for immunotherapy of cancer. Receptors of VISTA through which this protein transmits immunosuppressive signals under various normal and pathological conditions remain to be identified.

**Materials and methods:**

To conduct the study, we used human recombinant proteins and various human cell lines as well as primary T cells. A wide range of techniques including tissue culture and co-cultures, Western blot analysis, on-cell Western, ELISA, co-immunoprecipitation, biochemical assays and synchrotron radiation circular dichroism spectroscopy were employed.

**Results:**

Here we report for the first time that VISTA has affinity to programmed cell death protein 1 (PD-1) and binds it as a ligand. We found that when interacting with PD-1, VISTA suppresses interleukin 2 production by T helper cells. These effects were confirmed in the *in vitro* and *ex vivo* experiments. Affinity of VISTA to PD-1 was also characterised and found to be moderate, with a *K*_d_ of ∼2.3 μM detected by synchrotron radiation circular dichroism spectroscopy.

**Conclusions:**

These results open a completely new chapter in our understanding of the concept of immune checkpoint proteins, where some of them clearly show both ligand and receptor activities and display multifunctional properties.

## Highlights

• VISTA has affinity to PD-1 and binds it as a ligand.
• VISTA suppresses IL-2 production by T helper cells through PD-1.
• VISTA has both ligand and receptor activities and thus displays multifunctional properties.

## Introduction

V-domain immunoglobulin (Ig) -containing suppressor of T cell activation (VISTA) is a unique multifunctional immune checkpoint protein that was reported to show both receptor and ligand activities.^1^, ^2^, ^3^ Recent studies have revealed several ligands of VISTA, such as V-set and Ig domain containing 3 (VSIG-3) and galectin-9.^4^, ^5^, ^6^ These ligands are capable of suppressing the cytotoxic activities of T lymphocytes when binding to VISTA expressed on the cell surface. For example, galectin-9 changes plasma membrane potential when interacting with VISTA on the surface of T cells, thus preventing the release of granzyme B from these cells, where the resulting internal activation of granzyme B causes programmed death of T cells.^4^, ^5^, ^6^

Recently, the cell-surface protein P-selectin glycoprotein ligand-1 (PSGL-1) was discovered to recognise VISTA as its ligand within the malignant tumour microenvironment at acidic pH. PSGL-1-VISTA interactions lead to the suppression of T cell functions.^7^ However, soluble and cell-associated forms of VISTA were shown to suppress the activities of T cells under non-acidic pH conditions too, implying that there are other T cell receptors that recognise VISTA as a ligand.^8^^,^^9^ Importantly, when suppressing T cell functions, VISTA acts in a similar fashion to programmed death-ligand 1 (PD-L1) interacting with the receptor programmed cell death protein 1 (PD-1).^8^ However, *in vivo* studies indicated that VISTA-positive cancers were resistant to anti-PD-1 therapy.^8^^,^^10^ This indicates that VISTA either does not act through PD-1 or has affinity to several different types of inhibitory T cell receptors. Given the structural and functional similarities of VISTA and ligands of the immune checkpoint receptor PD-1, such as PD-L1 and PD-L2, we hypothesised that VISTA may display certain affinity to PD-1 and, as such, suppresses T cell functions in a PD-1-dependent manner. Understanding these mechanisms is crucial for developing immunotherapies against cancers that use VISTA as an immune checkpoint protein.^3^ This is potentially important given current challenges faced by immunotherapy of cancers, since immune checkpoint pathways form a complex machinery^11^ and understanding these cross-links is vital to develop highly efficient strategies for personalised and affordable immunotherapy of cancer. We therefore aimed to investigate whether VISTA suppresses T cell activities *via* PD-1. This would include activation of Src homology region 2-containing protein tyrosine phosphatase 2 (SHP-2) followed by downregulation of phosphatidylinositol-3-kinase (PI3K) and mitogen-activated protein kinase (MAPK) pathways. These effects lead to suppression of interleukin 2 (IL-2) production by helper T cells and the attenuation of antiapoptotic machinery in cytotoxic T cells. Furthermore, these actions of VISTA would kill cytotoxic T cells, rendering them unable to implement antiapoptotic machinery. Here we report for the first time that VISTA has affinity to PD-1 and binds to it as a ligand. When interacting with PD-1, VISTA suppresses IL-2 production by T helper cells. We also demonstrate that PD-1 is most likely not the only receptor on the surface of T lymphocytes that VISTA can recognise.

## Materials and methods

### Materials

Cell culture media, foetal bovine serum, supplements and basic laboratory chemicals were obtained from Sigma (Suffolk, UK). Microtiter plates for enzyme-linked immunosorbent assay (ELISA) were obtained from Nunc (Roskilde, Denmark). Mouse antibodies against PD-1 (monoclonal, 66220-1-Ig) and β-actin (monoclonal, 66009-1-Ig) and rabbit antibody against VISTA (polyclonal, 24849-1-AP) were purchased from Proteintech (Manchester, UK). Anti-CD3 (OKT3) mouse monoclonal and anti-VISTA (BLR035F) rabbit monoclonal antibodies were purchased from Abcam (Cambridge, UK). Goat anti-mouse (925-32210 and 926-68070) and anti-rabbit (926-3211 and 926-68071) secondary antibodies were obtained from Li-COR (Lincoln, Nebraska). ELISA-based assay kits for the detection of PD-L1 (DY156), VISTA (DY7126) and IL-2 (DY202) as well as carrier-free recombinant human VISTA, PD-1 and PD-L1 were purchased from Bio-Techne (R&D Systems, Abingdon, UK). All other chemicals used in this study were of the highest grade of purity and commercially available.

### Cell lines and primary human T cells

Cell lines used in this work were purchased from either the European Collection of Cell Cultures or the American Type Culture Collection. Cell lines were accompanied by authentication test certificates. Jurkat T and K562 cells were cultured in RPMI 1640 medium supplemented with 10% foetal bovine serum, penicillin (50 IU/ml), and streptomycin sulfate (50 μg/ml). LN18 human glioblastoma cells were cultured using DMEM medium supplemented with 10% foetal bovine serum, penicillin (50 IU/ml), and streptomycin sulfate (50 μg/ml). Primary human T cells were isolated as described previously.^6^^,^^12^ Briefly, primary human T cells were isolated from buffy coat blood (purchased from healthy donors undergoing routine blood donation), which was purchased from the National Health Blood and Transfusion Service following ethical approval (REC reference: 16-SS-033). Primary T cells were purified using a commercial T cell purification kit (EasySep Human T Cell Isolation Kit, StemCell Technologies, Cologne, Germany) according to the manufacturer’s protocol.

### Western blot analysis

Levels of PD-1 were analysed by Western blot and compared with the amounts of β-actin (protein loading control), as previously described. Li-Cor goat secondary antibodies conjugated with infrared fluorescent dyes were used as described in the manufacturer’s protocol for visualisation of specific target proteins (the Li-COR Odyssey imaging system was employed). Western blot data were quantitatively analysed using Image Studio software and values were subsequently normalised against those of β-actin.^9^

### Detection of PI3K activity

PI3K activity was detected using a spectrophotometric method based on analysis of substrate [PI(4,5)-diphosphate] phosphorylation, as previously described.^13^

### Enzyme-linked immunosorbent assays (ELISAs)

Levels of secreted IL-2 as well as cellular levels of PD-L1 and VISTA were measured by ELISA using R&D Systems kits according to manufacturer’s protocols.

### Co-immunoprecipitation assay

To verify the interactions of PD-1 and VISTA in a physiological environment, we pre-coated ELISA plates with anti-PD-L1 or anti-VISTA capture antibodies and blocked using 1% bovine serum albumin in phosphate-buffered saline (PBS). PD-L1 (4 ng) or VISTA (4 ng) were then immobilised on these plates, respectively. Control wells were treated with PBS alone. Following immobilisation, plates were washed with TBST buffer and Jurkat T cell lysates (containing PD-1) were loaded. After 2 h of incubation at room temperature, plates were washed with TBST buffer and PD-1 (if bound) was extracted using glycine-HCl buffer (pH 2.0) as described before. The extracts were subjected to Western blot analysis of PD-1 as described previously^3^ and above.

### Synchrotron radiation circular dichroism (SRCD) spectroscopy

Human recombinant VISTA and PD-1 complexes were characterised using SRCD spectroscopy at B23 beamline, Diamond Light Source (Didcot, UK). Circular dichroism (CD) measurements were carried out with 0.2 μg/ml of samples using a 1 cm path length cell, 6.3 mm aperture diameter and 520 μl capacity using B23 Module A end-station and Chirascan Plus CD spectrophotometer (Applied Photophysics Ltd, Leatherhead, UK) with a 1 nm increment, 1s integration time, 1 nm bandwidth at 20°C. The obtained results were analysed with the help of CDApps (Diamond Light Source Ltd, Didcot, UK) and OriginPro™ (OriginLab Corp, Northampton, MA, USA).^14^, ^15^, ^16^

### On-cell Western analysis

Cell-surface levels of PD-1 and VISTA protein were measured using on-cell Western analysis carried out using a Li-COR Odyssey imager and the assay was performed in line with manufacturer’s recommendations as previously described.^17^

### Analysis of SHP-2-like phosphatase activity

Activities of DiFMUP (6,8 difluoro-4-methylumbelliferyl phosphate)-specific phosphatases (herein labelled ‘SHP-2-like phosphatases’^18^) activities were analysed using a fluorometric assay kit (E12020, Thermo Fisher Scientific) according to the manufacturer’s instructions.

### Cell viability assay

Cell viability was assessed using an MTS (3-(4,5-dimethylthiazol-2-yl)-5-(3-carboxymethoxyphenyl)-2-(4-sulfophenyl)-2H-tetrazolium) assay kit (Promega, Madison, WI, USA) according to the manufacturer’s protocol.

### Statistical analysis

Each experiment was carried out at least four times and statistical analysis was conducted using a two-tailed Student’s *t*-test. Multiple comparisons were performed using analysis of variance. *Post hoc* Bonferroni correction was applied. Statistical probabilities were expressed as ∗ when *P* < 0.05; ∗∗, *P* < 0.01 and ∗∗∗ when *P* < 0.001.

## Results

**Figure 1:**
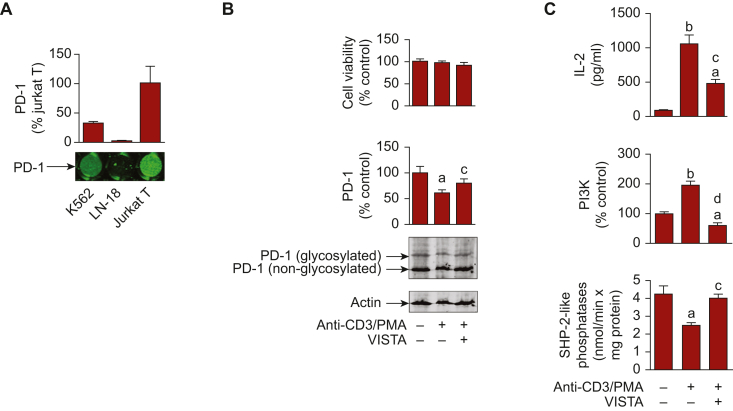

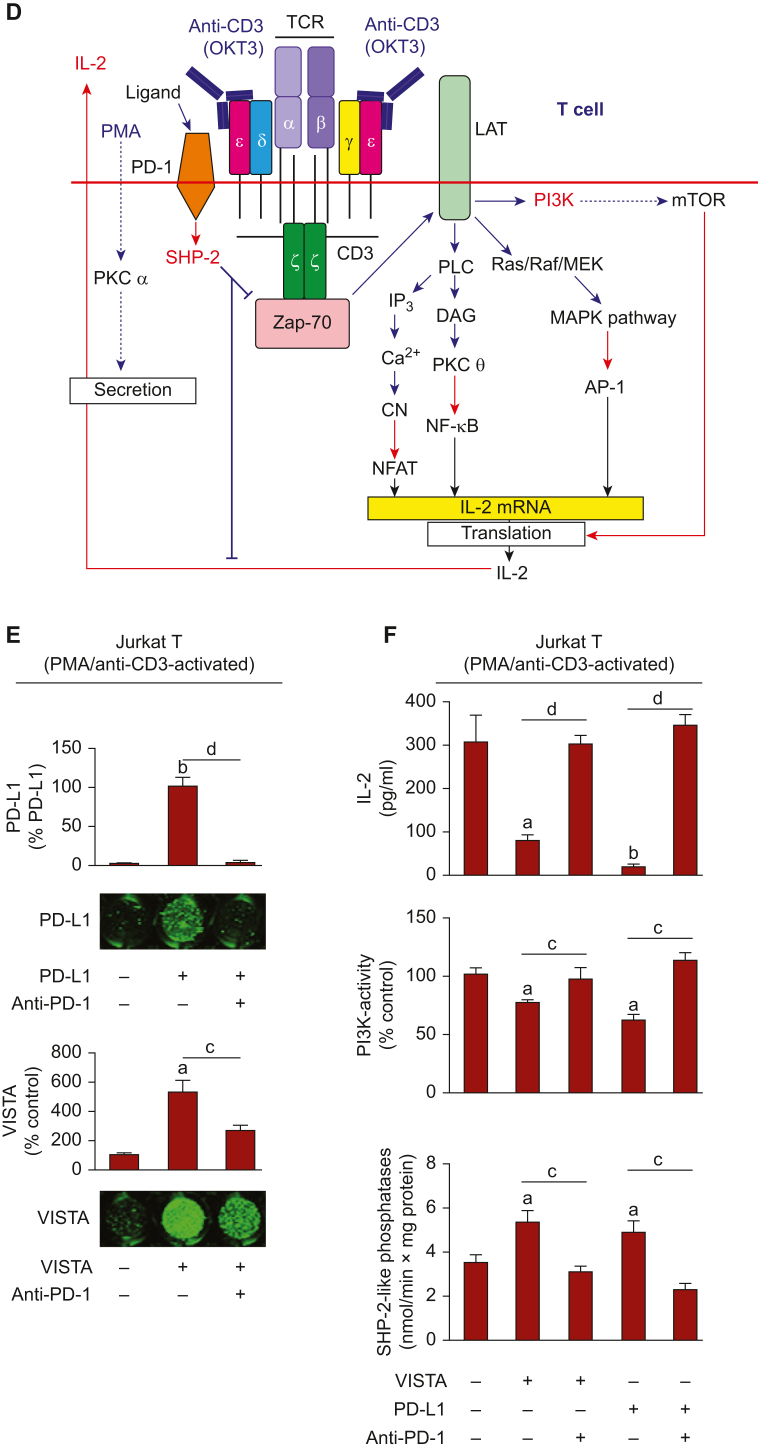
**VISTA downregulates anti-CD3/PMA-induced responses of Jurkat T cells in a PD-1-dependent manner.** (A) PD-1 shows high surface presence on Jurkat T cells compared with K562 and LN-18 cells as measured by on-cell Western analysis when analysing equal amounts of each cell type. (B) Jurkat T cells were exposed to 5 μg/ml anti-CD3 antibody in the presence of 100 nM PMA for 16 h in the absence or presence of 100 ng/ml VISTA. Cell viability and PD-1 expression (total levels including non-glycosylated and glycosylated proteins were quantified), as well as (C) activities of SHP-2-like phosphatases and PI3K and IL-2 release were analysed as outlined in Materials and Methods. (D) Mechanism of ligand-induced PD-1-mediated suppression of T cell functions. The mechanism underlying anti-CD3/PMA-induced IL-2 production is presented and it is demonstrated that PD-1 suppresses these effects in a ligand-dependent manner. (E) Jurkat T cells were exposed to 5 μg/ml anti-CD3 antibody in the presence of 100 nM PMA. In addition, we supplied 100 ng/ml of human recombinant VISTA or PD-L1 in the absence or presence of 5 μg/ml PD-1 neutralising antibody. Cell-surface levels of PD-L1 and VISTA were measured. Since PD-L1 was not detectable on the surface of resting Jurkat T cells, its levels were normalised against the sample, where cells were exposed to PD-L1, whereas VISTA levels were normalised against the control, where surface presence of VISTA was detectable. (F) Activities of PI3K and SHP-2-like phosphatases were measured in Jurkat T cell lysates and IL-2 levels were analysed in conditioned medium as outlined in the Materials and Methods. Images are from one experiment (representative of four, which gave similar results). Data are mean values ± SEM of four independent experiments. ^a^*P* < 0.05 and ^b^*P* < 0.01 versus control, ^c^*P* < 0.05 and ^d^*P* < 0.01 versus anti-CD3/PMA (panels B and C) or between indicated events (panels E and F). AP-1, activator protein 1; CN, calcineurin; IL-2, interleukin 2; NFAT, nuclear factor of activated T cells; NF-kB, nuclear factor kappa B; PD-1, programmed cell death protein 1; PD-L1, programmed death-ligand 1; PI3K, phosphatidylinositol-3-kinase; PMA, phorbol 12-myristate 13-acetate; SEM, standard error of means; SHP-2, Src homology region 2-containing protein tyrosine phosphatase 2; VISTA, V-domain immunoglobulin-containing suppressor of T cell activation. "+" under images and/or bar diagrams indicates presence of corresponding stimuli, and "−" indicates their absence.

To investigate VISTA-PD-1 interactions, we first used Jurkat T cells which predominantly display the properties of CD4+ T helper cells.^9^ We confirmed that PD-1 is present on the surface of Jurkat T cells using K562 human chronic myeloid leukaemia and LN-18 human glioblastoma cell lines, as positive and negative controls, respectively, using on-cell Western analysis (Figure 1A). We exposed the cells to 5 μg/ml anti-CD3 antibody in the presence of 100 nM phorbol 12-myristate 13-acetate (PMA) for 16 h in the absence or presence of 100 ng/ml VISTA. We observed that anti-CD3/PMA-induced activation of Jurkat T cells led to a moderate decrease in PD-1 expression and did not significantly influence T cell viability (Figure 1B). This stimulation also reduced the activity of SHP-2-like phosphatases and, respectively, upregulated the activity of PI3K and IL-2 production/secretion (Figure 1C). VISTA attenuated these effects, restoring PD-1 expression (Figure 1B) and downregulating IL-2 production as well as corresponding upstream pathways (Figure 1C). To run the investigation, we activated Jurkat T cells with 5 μg/ml anti-CD3 antibody in the presence of 100 nM PMA for 16 h with or without 30 min pre-exposure to 5 μg/ml PD-1 neutralising antibody. PD-L1 or VISTA (100 ng/ml) were added to some of the cell cultures simultaneously with anti-CD3/PMA to assess the suppressive activity of these immune checkpoint proteins. Anti-CD3 induces activation of zeta-chain-associated protein kinase 70 (Zap-70), which triggers activation of PI3K, MAPK and phospholipase C/protein kinase C θ (PKC-θ) pathways. These pathways activate transcription factors required to induce IL-2 expression.^19^, ^20^, ^21^ PMA triggers the activation of PKC-α and thus facilitates exocytosis^21^^,^^22^ (Figure 1D). Ligand-activated PD-1 is known to suppress PI3K and MAPK pathways by activating SHP-2. SHP-2 dephosphorylates T cell kinases like Zap-70, IL-2-inducible T cell kinase and CD28, which results in the inactivation of PI3K, MAPK and other pathways, and inhibition of IL-2 production, a cytokine that is required for cytotoxic T cell activation.^23^ When Jurkat T cells were exposed to PD-L1, it was clearly incorporated on the cell surface but was attenuated in the presence of PD-1 neutralising antibody (Figure 1E). The same pattern was observed with VISTA (Figure 1E). Importantly, pre-exposure of Jurkat T cells to anti-PD-1 reduced, but did not attenuate, VISTA interaction with the surface of Jurkat T cells, suggesting that VISTA possibly binds to PD-1 as well as other cell-surface receptors (Figure 1E).

We next asked whether the possible VISTA-PD-1 interaction impacted on the helper activities of Jurkat T cells, associated with IL-2 production/secretion. In anti-CD3/PMA-activated Jurkat T cells exposed to VISTA or PD-L1, the activities of SHP-2-like phosphatases^18^ were significantly upregulated compared with anti-CD3/PMA-activated cells alone. PI3K activity and IL-2 secretion were both downregulated (Figure 1F). When cells were pre-exposed to anti-PD-1, the effects outlined above were downregulated but not attenuated (Figure 1F).

**Figure 2:**
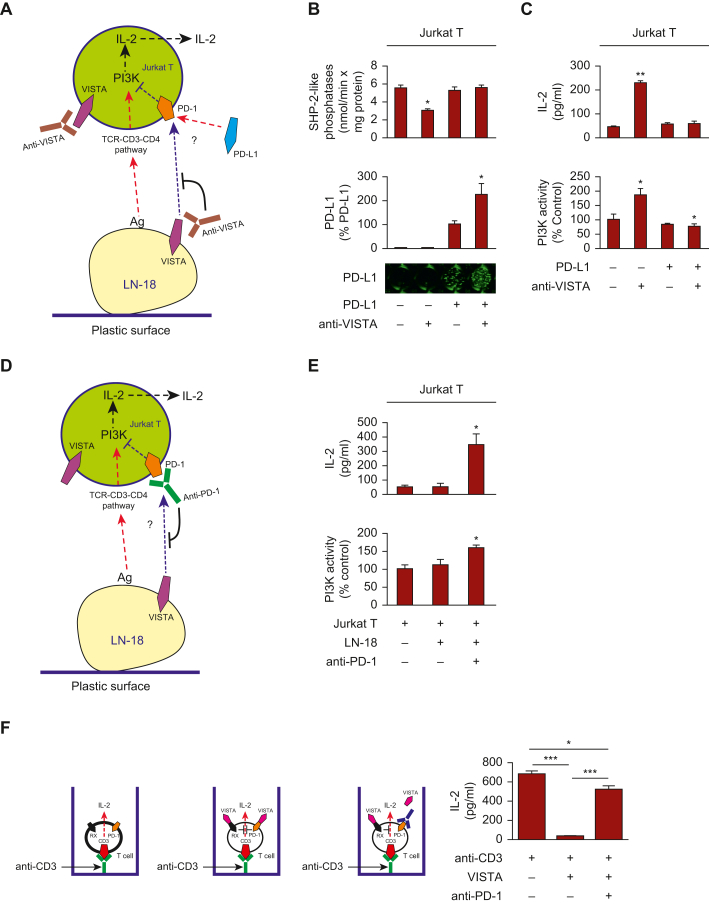
**Cell-associated and soluble forms of VISTA downregulate T cell responses through PD-1.** (A) LN-18 cells, which express VISTA on the cell surface, were co-cultured with Jurkat T cells at a ratio of 1 : 1 for 16 h in the absence or presence of 5 μg/ml anti-VISTA antibody and/or 100 ng/ml PD-L1. (B) Jurkat T cells were then removed and subjected to analysis of PD-L1 cell-surface presence and SHP-2-like phosphatase activity. (C) PI3K activity was measured in Jurkat T cell lysates and IL-2 activity in conditioned medium. (D) LN-18 cells were co-cultured with Jurkat T cells at a ratio 1 : 1 for 16 h in the absence or presence of PD-1 neutralising antibody. (E) PI3K activity was analysed in Jurkat T cell lysates and IL-2 levels were measured in the conditioned medium. (F) Primary human T cells were immobilised on plates pre-coated with anti-CD3 antibody that also acts as a stimulus (assessed by measurement of IL-2 production). 100 ng/ml VISTA was added to the indicated wells with or without supplying 5 μg/ml anti-PD-1 antibody. IL-2 release was then analysed by ELISA in the conditioned medium. Images are from one experiment (representative of four, which gave similar results). Data are mean values ± SEM of four independent experiments. ∗*P* < 0.05, ∗∗*P* < 0.01, and ∗∗∗*P* < 0.001 versus control or indicated events. ELISA, enzyme-linked immunosorbent assay; IL-2, interleukin 2; PD-1, programmed cell death protein 1; PD-L1, programmed death-ligand 1; PI3K, phosphatidylinositol-3-kinase; SEM, standard error of means; SHP-2, Src homology region 2-containing protein tyrosine phosphatase 2; TCR, T cell receptor; VISTA, V-domain immunoglobulin-containing suppressor of T cell activation. "+" under images and/or bar diagrams indicates presence of corresponding stimuli, and "−" indicates their absence.

Since these experiments were conducted with human recombinant VISTA, we were interested to see if the same effects could be achieved with cell surface-expressed VISTA. From our previous work we know that VISTA is present on the surface of LN-18 human high-grade glioblastoma cells.^9^ We therefore co-cultured LN-18 cells with Jurkat T cells (at a ratio of 1 : 1) for 16 h in the absence or presence of 100 ng/ml PD-L1 and/or 5 μg/ml VISTA-neutralising antibody^9^ (Figure 2A). Here, VISTA neutralisation downregulated SHP-2-like phosphatase activity but the presence of PD-L1 restored their original activities. Interestingly, when VISTA was neutralised and PD-L1 was present, cell-surface levels of PD-L1 were upregulated compared with when VISTA was active and PD-L1 was present. This suggests a competition between VISTA and PD-L1 for the same receptor (Figure 2B). Similar responses were observed for PI3K and IL-2 secretion, where an upregulation of both were observed upon VISTA neutralisation and a downregulation by PD-L1 regardless of the presence of VISTA-neutralising antibody. This suggests that PD-L1 was just ‘covering for’ VISTA, when VISTA was neutralised, otherwise an enhanced effect would have been seen (Figure 2C).

To verify that the effects observed were PD-1-dependent, we co-cultured LN-18 and Jurkat T cells at a ratio of 1 : 1 for 16 h in the absence or presence of 5 μg/ml of PD-1-neutralising antibody (Figure 2D). We assessed PI3K activity in Jurkat T cell lysates and IL-2 levels in conditioned medium and found that PD-1 neutralisation led to upregulation of both PI3K activity and IL-2 release, indicating the role of PD-1 in suppression of these effects (Figure 2E).

Based on these results we sought to obtain the proof of principle in primary T cells. Primary T cells were captured on 96-well MaxiSorp plates (Nunc, Roskilde, Denmark), using immobilised anti-CD3 (anti-CD3 immobilised on the surface can induce IL-2 production/secretion in T cells in the absence of PMA), and incubated for 24 h (so that anti-CD3 could stimulate T cell IL-2 production) in the absence or presence of 100 ng/ml human recombinant VISTA, with or without PD-1 neutralising antibody (Figure 2F). This experiment revealed that VISTA attenuates T cell IL-2 production, whereas the presence of anti-PD-1 antibody increases but does not completely restore IL-2 secretion (Figure 2F).

**Figure 3:**
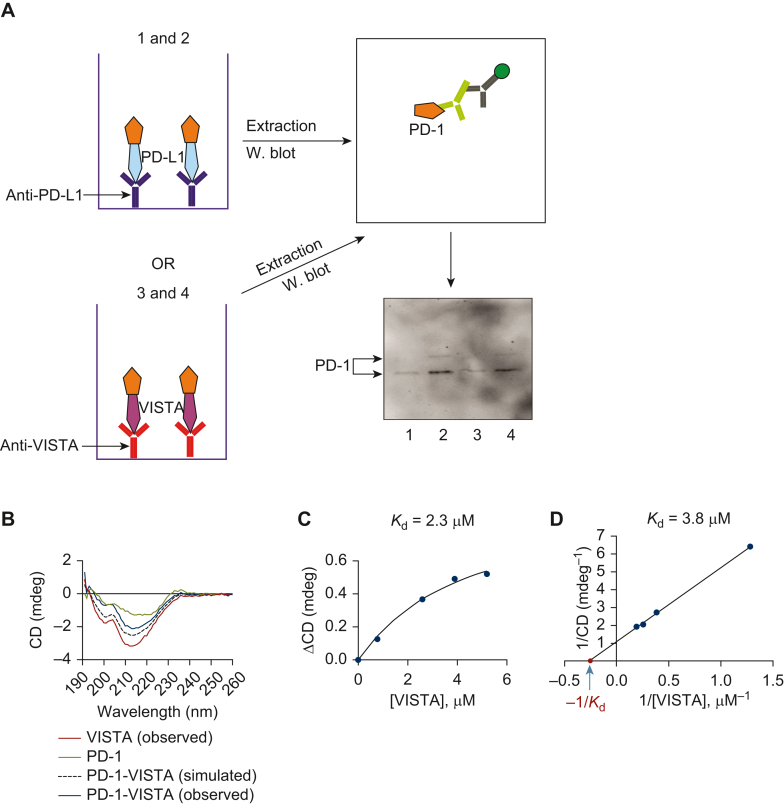
**Characterisation of interactions between human VISTA and PD-1.** (A) ELISA 96-well plates were pre-coated with anti-PD-L1 or anti-VISTA antibodies as outlined in the Materials and Methods and human recombinant PD-L1 or VISTA were immobilised through these antibodies. Samples were then incubated with PD-1-containing Jurkat T cell lysates for 2 h followed by extraction of bound PD-1 and its detection by Western blot. The left panel shows the scheme of co-immunoprecipitation; the right panel, Western blot analysis of extracted proteins from the plate (scheme on the top and results at the bottom). Lane 1: extracts from wells coated with anti-PD-L1 capture antibody followed by exposure to Jurkat T cell lysates; lane 2: extracts from wells coated with anti-PD-L1 capture antibody, followed by immobilisation of human recombinant PD-L1 on it followed by exposure to Jurkat T cell lysates; lane 3: extracts from wells coated with anti-VISTA capture antibody followed by exposure to Jurkat T cell lysates; lane 4: extracts from wells coated with VISTA capture antibody, followed by immobilisation of human recombinant VISTA on it, followed by exposure to Jurkat T cell lysates. Images are from one experiment (representative of three, which gave similar results). (B-D) Human recombinant carrier-free VISTA and PD-1 were subjected to analysis of specific interactions using SRCD spectroscopy. (B) assessment of binding; (C) titration for the purpose of *K*_d_ calculations using Hill plot; (D) calculation of *K*_d_ using Lineweaver-Burk plot. Data are mean values of four independent experiments. ELISA, enzyme-linked immunosorbent assay; PD-1, programmed cell death protein 1; PD-L1, programmed death ligand 1; SRCD, synchrotron radiation circular dichroism; VISTA, V-domain immunoglobulin-containing suppressor of T cell activation, W, Western.

To verify binding of VISTA to PD-1 we pre-coated 96-well MaxiSorp plates with anti-PD-L1 or anti-VISTA antibodies and then immobilised human recombinant PD-L1 or VISTA through these antibodies. We then applied Jurkat T cell lysates to the wells and subsequently extracted bound proteins after 2 h incubation as described before.^3^ The extracts were then loaded onto the gels and subjected to Western blot analysis of PD-1. For controls we used wells not containing either PD-L1 or VISTA. We found that PD-1 was highly present in the samples where PD-L1 or VISTA were immobilised (Figure 3A). In samples containing Jurkat T cell lysates alone, we saw traces of PD-1, suggesting the appearance of PD-1-VISTA and PD-L1-PD-1 complexes formed by proteins expressed by Jurkat T cells (Figure 3A), which express both PD-L1 (1980 ± 196 pg/mg protein) and VISTA (2790 ± 412 pg/mg protein) as measured by ELISA.

Finally, we assessed the direct interactions of PD-1 and VISTA using recombinant proteins. For this purpose, we used CD spectroscopy. Based on the CD spectra, we saw a clear specific interaction between VISTA and PD-1 (Figure 3B). Titration experiments with a single concentration of PD-1 and increasing concentrations of VISTA revealed that the affinity constant (*K*_d_) of VISTA to PD-1 is 2.3 μM when a Hill plot is applied (Figure 3C). If a Lineweaver-Burk type plot is used, the calculated *K*_d_ = 3.8 μM (Figure 3D).

## Discussion

In this work we investigated whether VISTA can interact with PD-1 when acting as a ligand. Firstly, we confirmed that VISTA suppresses helper T cell function (a test that is currently used to verify VISTA ligand activity^9^). We then showed that neutralisation of PD-1 can impair the ability of VISTA to interact with cell-surface receptors and restores T cell activities (in particular, IL-2 production, see Figures 1 and 2). This was verified for both human recombinant VISTA and VISTA protein displayed as immune checkpoint on the surface of human high-grade glioblastoma cells (Figures 1 and 2). The effects of VISTA and PD-1 neutralisation, as a way to downregulate but not attenuate VISTA immunosuppressive effects, was also verified in *ex vivo* experiments using primary human T cells (Figure 2D). Our results showed that VISTA most likely recognises and engages in interactions with several receptors on the T cell surface and PD-1 is definitely one of them. Given that specific and biologically efficient interactions between VISTA and PD-1 were confirmed directly using several approaches including co-immunoprecipitation, we also studied the affinity of these proteins to each other using SRCD spectroscopy. This method appears to be more reliable, since it does not require immobilisation of one of the proteins of the surface and, as such, rules out a possibility of false-negative results. We found that there was moderate affinity between VISTA and PD-1 (the *K*_d_ was between 2.3 and 3.8 μM depending on the approach used for calculation). This is similar to the one reported for PD-L1 versus PD-1 (*K*_d_ 8.2 μM), which, however, was measured using surface plasmon resonance.^24^^,^^25^ Conversely, it is quite moderate compared, for example, with VISTA affinity to galectin-9 measured by SRCD spectroscopy (with a *K*_d_ of 18 nM^6^). SRCD spectroscopy demonstrated conformational changes which both VISTA and PD-1 undergo during the interaction (Figure 3B) and based on these observations and *in vitro/ex vivo* experiments presented in the Figures 1 and 2, these interactions are sufficient to cause immunosuppressive biological responses. It is, however, important to stress that further investigations are required to study how VISTA/PD-1 interactions impact on the anticancer activities of cytotoxic T cells and *in vivo* immunosuppression. This will help to determine optimal targets to pharmacologically correct this immune checkpoint pathway with the purpose of developing highly efficient strategies for immunotherapy of VISTA-dependent cancers.

Taken together, these results open a completely new direction in our understanding of the concept of immune checkpoints, where some of them clearly display both ligand and receptor properties. And given the level of affinity to receptors (e.g. VISTA to PD-1, PD-L1/PD-L2 to PD-1),^24^^,^^25^ it is highly likely that each of the checkpoints has several receptors. Since these are moderate affinity (compared for example with affinities of antigen-antibody interactions) but nonetheless biologically efficient interactions, presumably such immune checkpoints as VISTA and PD-1 can interact with several proteins (receptors or ligands). VISTA is a notable example of this, since it can interact with PSGL-1^7^ as well as with PD-1 as a ligand and with galectin-9 and VSIG-3 as a receptor.^3^, ^4^, ^5^, ^6^ From our results it is evident that VISTA can interact with even more receptors on the surface of T cells, since PD-1 neutralisation does not completely attenuate VISTA effects and our experiments were performed at neutral pH, where VISTA cannot interact with PSGL-1.^7^ Therefore, this work shows that the concept of immune checkpoints is much more complex compared with what it is currently considered to be.
